# Cannulated prolactin test: Useful test to investigate patients with mild hyperprolactinemia?

**DOI:** 10.17925/EE.2026.22.2.4

**Published:** 2026-08-04

**Authors:** Tânia Matos, Daniela Dias, Inês Sapinho, Pedro Marques

**Affiliations:** 1. Endocrinology Department, Hospital CUF Descobertas, Lisbon, Portugal; 2. Faculty of Medicine, Universidade Católica Portuguesa, Lisbon, Portugal

**Keywords:** Cannulated prolactin, hyperprolactinemia, prolactin, prolactinoma, stress-induced hyperprolactinemia, venipuncture stress

## Abstract

We discuss the highlights of Matos et al., 2026, publication, which explores the usefulness of the cannulated prolactin test (CPT) when assessing patients with mild hyperprolactinemia. The publication presents data from their own cohort, alongside a pooled analysis of eleven available published series, combining a total of 1,700 patients. The findings support the CPT role in the investigation of mild hyperprolactinemia patients and propose a stepwise approach in which prolactin should be first repeated under optimal (stress-free) conditions, reserving full CPT for those with persistent hyperprolactinemia.

Hyperprolactinemia is a frequent cause for medical referrals to endocrinology departments. Markedly elevated prolactin levels are generally indicative of macroprolactinomas, whereas mild to moderate hyperprolactinemia may arise from physiological factors (such as exercise, sleep or stress), pharmacological agents, metabolic disturbances, untreated primary hypothyroidism, as well as hypothalamic–pituitary disorders, including microprolactinomas or pituitary stalk compression due to pituitary or parasellar lesions.^1–5^ Thus, the differential diagnosis between pathological hyperprolactinemia and mild hyperprolactinemia due to stress/physiological causes is critical to avoid unnecessary diagnostic investigations, misdiagnosis or inadequate treatment.^1^

Performing a cannulated prolactin test (CPT), where serial prolactin sampling is collected over a period of 60–120 min in a controlled and stress-free environment, has the potential to help distinguish patients with elevated prolactin levels due to venipuncture stress from those with pathological hyperprolactinemia. However, the current data are limited, and the few individual series are heterogeneous and generally involve a small cohort of cases; hence, the usefulness of CPT remains controversial and not widely recognized.^2^ Therefore, we recently conducted a retrospective study on our cohort of 105 patients with mild hyperprolactinemia (less than five times the upper limit of normal) who underwent CPT, as well as a systematic review and pooled analysis of the cases previously published in the literature. These data are now published in the journal *Pituitary*.^6^

Overall, our data supported the usefulness of CPT in excluding pathological hyperprolactinemia in individuals with mild hyperprolactinemia. In our series, the prolactin normalization rate was 59%. When combined with previously published series, the pooled analysis encompassing a total of 1,700 patients (86.8% females) indicated an overall prolactin normalization rate of 53.7%. These results show that pathological hyperprolactinemia may be ruled out by CPT in more than 50% of patients with mild elevations of serum prolactin, who otherwise may undergo unnecessary imaging and eventually inadequate treatment with dopamine agonists if a pituitary adenoma is incidentally identified. We also found that about 75% of patients undergoing CPT have normal prolactin levels at the test baseline. There were no differences in prolactin normalization rates between females or males. These findings highlight the importance of repeating prolactin sampling in patients with mild hyperprolactinemia in a stepwise approach, in which prolactin is first repeated under stress-free conditions, with the CPT reserved for individuals with persistent hyperprolactinemia.

Prolactin has a pulsatile secretion; yet, as previously discussed, painful venipuncture, psychological and anticipatory stress may also contribute to transitory prolactin elevations.^1,2,7,8^ We found that three-quarters of patients undergoing CPT actually have normal prolactin at CPT baseline (PRL0′). This may reflect the controlled and low-stress conditions under which CPT is performed, with blood sampling obtained after catheter placement. Consequently, ensuring prolactin assessment in a calm, stress-minimized setting may be sufficient to prevent unnecessary CPT in a substantial proportion of patients.

Interestingly, in our case series, among the 15 patients with elevated PRL0′, 10 normalized prolactin at 30 min (66.7%) and 5 patients at 60 min (33.3%) during CPT. Similar normalization patterns have been reported in other studies with heterogeneous protocols ranging from 30 to 120 min, while protocols shorter than 20–30 min may be inadequate given the prolactin half-life of 20–50 min. Prolonging the CPT beyond 60 min provides minimal additional detection but increases patient burden and healthcare costs. Therefore, a 60 min CPT offers an effective and practical compromise between sensitivity, patient comfort and resource utilization.

Our publication also highlights the dangers of overdiagnosing. Within our series, 15 patients with prolactin normalization during CPT still underwent magnetic resonance imaging, and a pituitary microadenoma was found in seven (47%) of them (i.e. a pituitary incidentaloma), leading to increased risk of misdiagnosing a microprolactinoma and starting dopamine agonist treatment inappropriately. These data reinforce further the advantages of performing a CPT in individuals with mild hyperprolactinemia, which relates to the reduction in unnecessary investigations, including the detection of pituitary incidentalomas, and treatment mismanagement, while potentially allowing for a reduction in healthcare costs, patient burden and anxiety.

Despite the CPT’s potential role and usefulness in clinical practice, the small number of publications, the discrepant data and the absence of proposed cut-offs and sampling protocols contribute to its reduced use in clinical practice. These authors’ publication, combining the results of 1,700 CPTs, represents an important step to provide further insights into this matter, reinforcing CPT as a tool in patients who present with persistent hyperprolactinemia after serum prolactin measurement repetition under optimal and stress-free conditions. Nevertheless, prospective, well-controlled and large studies are needed to establish the normal ranges for prolactin during CPT and to define the best and most cost-effective protocol for CPT, including the best duration for prolactin sampling during the CPT.
